# Recurrent *Clostridium difficile* infection treated with home fecal transplantation: a case report

**DOI:** 10.1186/1752-1947-8-393

**Published:** 2014-11-28

**Authors:** Pauline S Duke, John Fardy

**Affiliations:** 19 Discipline of Family Medicine, Faculty of Medicine, Memorial University, 300 Prince Phillip Drive, St. John’s, NF A1B 3V6 Canada; 20 Discipline of Medicine, Faculty of Medicine, Memorial University, 300 Prince Phillip Drive, St. John’s, NF A1B 3V6 Canada

**Keywords:** *Clostridium difficile* infection, Fecal transplantation, Healthcare resources

## Abstract

**Introduction:**

*Clostridium difficile* infection causes severe diarrhea, abdominal pain and weight loss. A course of metronidazole is the initial treatment; however up to 40% of patients have at least one recurrence. Some patients have recurrent infections requiring further treatment with vancomycin, others need multiple courses of expensive treatment. Fecal transplantation has been proposed as an effective treatment option for patients with recurrences. We report the case of a patient with recurrent *Clostridium difficile* infection unresponsive to usual treatment and her experience with home fecal transplantation.

**Case presentation:**

A 66-year-old Canadian Caucasian woman presented to her family doctor in December 2012 with a 10-day history of explosive watery diarrhea. She was diagnosed with *Clostridium difficile* infection and treated with metronidazole. Diarrhea recurred and despite treatment with vancomycin and finally, fidaxomicin, she continued to have recurrent *Clostridium difficile* infection over the following four months. A formal fecal transplantation program was not available in her home province; therefore home fecal transplantation was performed under supervision by her family physician. This was the first case of fecal transplantation performed in the province and was done outside of a hospital setting. She recovered immediately and has been well for the past year since the procedure.

**Conclusions:**

Home fecal transplantation by rectal enema is a viable, safe and practical option for patients with recurrent *Clostridium difficile* infection. It is less costly and uses fewer resources than traditional delivery methods through nasogastric tube, upper endoscopy or colonoscopy. Patients and their families and donors need medical supervision through the process of screening, telephone availability during the procedure and medical follow-up. This can be done by family physicians without the need for expensive hospital care and subsequent follow-up.

## Introduction

*Clostridium difficile* infection (CDI) causes severe diarrhea, abdominal pain and weight loss. A course of metronidazole is the recommended initial treatment; however, up to 40% of patients have at least one recurrence [1]. Some patients have recurrent infections requiring further treatment with vancomycin, others need multiple courses of expensive treatment. Fecal transplantation has been proposed as an effective treatment option for patients with recurrences. Here we report the case of a patient with recurrent CDI unresponsive to usual treatment and her experience with home fecal transplantation.

## Case presentation

A 66-year-old Canadian Caucasian woman presented to her family doctor in December 2012 with a 10-day history of explosive watery diarrhea. This was accompanied by an initial fever and chills for two days, abdominal cramping, nausea and poor appetite but no rectal bleeding. There had been an 8kg weight loss over the previous four months. There was no history of recent travel or infectious contacts; however she had made weekly visits to a nursing home for some months prior. Amoxicillin was prescribed for a dental infection four months previously. Approximately 14 months and again at nine months prior to this present episode, she had bouts of diarrhea lasting about one week. Past medical and family history was noncontributory. Medications included atorvastatin and diclofenac sodium/misoprostol. She looked thin and her examination showed normal vital signs, no fever and generalized abdominal tenderness, worse in the left lower quadrant. A further general physical exam was negative for other sources of infection. Lab investigations showed normal results for complete blood count, electrolytes, blood urea nitrogen, creatinine, estimated glomerular filtration rate, glucose and liver function. Stools for culture and sensitivity and *C. difficile* toxin were requested. The differential diagnosis included an infectious etiology, inflammatory bowel disease or CDI. Ciprofloxacin and metronidazole were started but ciprofloxacin was discontinued following a negative sigmoidoscopy and positive *C. difficile* toxin test result the following day. Metronidazole was continued for 10 days along with probiotics, but severe diarrhea returned three days after finishing. Severe diarrhea recurred despite further treatment with vancomycin. Her repeat blood tests were normal apart from an increased serum calcium level of 2.7mmol/L (normal range: 2.10 to 2.55mmol/L). Her repeat *C. difficile* toxin test was again positive. Fidaxomicin 200mg orally twice a day was prescribed for 10 days, however symptoms recurred. Vancomycin was restarted while arrangements were investigated for out-of-province treatment with fecal transplantation.

Our patient and her husband asked their family doctor about home fecal transplantation, and had with them a copy of a journal article on home fecal transplantation by Silverman *et al*. [1]. The family doctor contacted the lead author by phone for further discussion and details about the procedure. Our patient’s healthy daughter was selected as donor, and her husband was screened in the event that another donor was required. Screening tests suggested by Silverman *et al*. are listed in Table 1.

**Table 1:** **List of screening tests for potential donors and recipients prior to fecal transplantation**

Screening type	
Screening serology (donor)	Human immunodeficiency virus (HIV)
	Human T-lymphotropic virus I/II
	Syphilis enzyme immunoassay
	Hepatitis A immunoglobulin M
	Hepatitis B surface antigen
	Hepatitis C antibody
	*Helicobacter pylori* antibody
Screening serology (recipient)	Complete blood count
	Sequential multi-channel analysis with computer-20 (Chem-20)
	Serum protein electrophoresis
	Serum immunoglobulins
	HIV antibodies
	Antigliadin antibodies
Stool testing (donor and recipient)	Culture and sensitivity
	Ova
	Parasites (three separate specimens)
	Cryptosporidia
	Microspora
	*Clostridium difficile* toxin

List from Silverman *et al*. [1].

Home fecal transplant, using the protocol recommended by Silverman *et al*. [1], was done within a week, with the assistance of our patient’s husband and daughter. Vancomycin was stopped for 48 hours prior to the procedure and *Saccharomyces boulardii* was taken during the transplant and for 60 days after. Donor stool in the amount of 50ml, obtained immediately prior to the procedure, was added to 200ml of normal saline in a blender. The contents were blended to a ‘milkshake’ consistency and emptied into an enema bag. The mixture was administered by her family and she lay supine on her left side for five hours afterwards to prevent defecation.

Diarrhea recurred and so the transplant was repeated on day three, with her lying supine for seven hours (two hours longer than with the previous procedure). The diarrhea was resolved immediately, however both our patient and her family decided to repeat the transplant using her husband as the donor two weeks later when the symptoms returned. There was immediate resolution of the diarrhea with no further recurrence of symptoms. Her appetite and energy returned and she had gained 15lbs two months after the transplant.

She underwent further investigations for hypercalcemia during this time and subsequently underwent a parathyroidectomy for removal of a parathyroid adenoma.

## Discussion

CDI is thought to result from the use of antibiotics that alter normal bowel flora which allows infection with pathogenic strains of *C. difficile*[2]. Exposure to a healthcare setting, the use of gastric acid-suppressing medications and underlying bowel conditions have all been established as potential risk factors [3]. In the United States, the incidence of CDI tripled between 1996 and 2005 (31 per 100,000 versus 84 per 100,000). There has also been an increase in disease severity, with mortality rates of up to 6.9% [4]. Failure rates with metronidazole treatment have risen from 2.5% to more than 18% since 2000 [4]. Patients older than 65 have a recurrence rate of up to 50%, increasing to greater than 60% after two episodes [4]. Many patients require multiple courses of antibiotics such as metronidazole and vancomycin. Fidaxomicin, an additional treatment option, is extremely costly. Hence, clinicians are interested in finding alternative treatments which are more successful and less expensive for the patient.

Fecal transplantation or fecal bacteriotherapy was first documented in 1958 [2]. The procedure involves transplantation of donor stool to the affected patient by various methods such as nasogastric tube, colonoscopy, rectal catheter or enema to repopulate the bowel with normal flora. The literature suggests that fecal transplantation has an extremely high cure rate, with recent systematic reviews reporting resolution of symptoms that ranged from 81 to 93% depending on the site and method of administration [5, 6]. Procedures where donors were family members had a higher success rate than nonrelated donors (93 versus 84%) [2], and procedures administering donor feces to the cecum and distal colon have a higher cure rate than the duodenum, stomach or jejunum (93 versus 81 to 86%) [5].

A Canadian study by Kassam *et al*. reviewed 27 patients with recurrent CDI treated with fecal transplantation by retention enema [4]. In this series, 93% of patients had resolution of their symptoms and five patients needed a second transplant due to recurrence, with three of these having full resolution. There were no adverse events or complications associated with the procedure. Another Canadian study by Silverman *et al*. followed seven patients who had self-administered home fecal transplantation. Patients ranging in age from 30 to 88-years-old had recurrent CDI, with the initial infection occurring in hospital. All donors were family members. The procedure was carried out by the patient or family members and all patients had resolution of CDI [1]. In a follow-up systematic review and meta-analysis, Kassam *et al*. found that lower gastrointestinal delivery of fecal transplant (for example by enema or colonoscopy) has a higher resolution rate than upper gastrointestinal delivery (for example nasogastric or nasojejunal tube or gastroscopy) (Table 2) [6].

**Table 2:** **Resolution rate for** ***Clostridium difficile*** **infection treated with fecal transplant by method of delivery**

Method of delivery	Resolution rate n/N (%) (unweighted)
Lower gastrointestinal delivery (colonoscopy, enema)	203/222 (91.4%)
Upper gastrointestinal delivery (nasogastric or nasojejunal tube, gastroscopy, gastrostomy tube)	42/51 (82.3%)

Adapted from Kassam *et al*. [6].

Our patient was very accepting of home fecal transplantation as there was no formal fecal transplantation program in the province. She had the help of family to administer the procedure and to be donors. They reported that, in their experience, it would be impossible for a patient to carry out the procedure without help from family or friends. Patients and families need help in obtaining the small amount of equipment needed and need medical supervision and support throughout the process, telephone availability during the procedure and medical follow-up.

Home fecal transplantation is less costly than multiple courses of expensive drugs. There may be fewer adverse effects [7] and less use of hospital resources with rectal enema than delivery through nasogastric tube, endoscopy or colonoscopy. Health authorities may consider using rectal enemas in a home or hospital-based fecal transplantation program for patients who would find it difficult to carry out this procedure on their own at home.

Our patient found the article by Silverman *et al*. [1] online in a search for alternatives to treat her recurrent severe CDI. This highlights the importance of patients’ participation and autonomy in their own care and the need for patient-centered care in the medical care of patients and their families.

We do not have a fecal transplantation program available in our home province of Newfoundland and Labrador (population of 526,000) in Canada. The closest program is in Ontario, over 3000 kilometers from Newfoundland. Newfoundland is an island; seeking treatment in Ontario would entail great travel and accommodation costs for her and her husband, as well as extreme discomfort when still feeling very ill from the infection. By doing this procedure at home by rectal enema, we prevented out-of-province travel and further costs for our patient.

## Conclusions

Home fecal transplantation by rectal enema is a viable, safe and practical option for patients with recurrent CDI. It is less costly and uses fewer resources than traditional delivery methods through nasojejunal tube, upper endoscopy or colonoscopy. Patients and their families and donors need medical supervision throughout the process of screening, telephone availability during the procedure and medical follow-up.

## Consent

Written informed consent was obtained from the patient for publication of this case report. A copy of the written consent is available for review by the Editor-in-Chief of this journal.

## Authors’ information

PD is a family physician and Professor in the Discipline of Family Medicine, Faculty of Medicine, Memorial University. JF is a Professor of Medicine (Gastroenterology) with Memorial University and Division Chief, Gastroenterology, Eastern Health. Both live in St. John’s, Newfoundland, Canada.

## Abbreviations

CDI: *Clostridium difficile* infection.
